# Application of the mineral-binding protein fetuin-A for the detection of calcified lesions

**DOI:** 10.7150/thno.78773

**Published:** 2023-01-01

**Authors:** Robert Dzhanaev, Christian Hasberg, Andrea Gorgels, Carlo Schmitz, Camilla Franziska Winkler, Hanna Malyaran, Steffen Gräber, Anouk Gentier, Armand Jaminon, Stijn Agten, Tilman Hackeng, Asim Cengiz Akbulut, Leon Schurgers, Felix Manuel Mottaghy, Claudia Goettsch, Willi Jahnen-Dechent

**Affiliations:** 1 Helmholtz-Institute for Biomedical Engineering, RWTH Aachen University Hospital, Aachen, Germany.; 2 IZKF - Interdisciplinary Center for Clinical Research, RWTH Aachen University Hospital, Aachen, Germany.; 3 Department of Biochemistry, Cardiovascular Research Institute Maastricht, Maastricht University Medical Centre, Maastricht, The Netherlands.; 4 Department of Nuclear Medicine, University Hospital Aachen, RWTH Aachen University, Aachen, Germany.; 5 Department of Radiology and Nuclear Medicine, Maastricht University Medical Centre, Maastricht, The Netherlands.; 6 Department of Internal Medicine I, Cardiology, Medical Faculty, RWTH Aachen University Hospital, Aachen, Germany.

**Keywords:** ectopic calcification, fetuin-A, calcification imaging, fluorescent proteins

## Abstract

**Rationale:** Calcium plays an essential role in the biology of vertebrates. Calcium content in body fluids is maintained within a narrow physiologic range by feedback control. Phosphate is equally important for metabolism and is likewise controlled, albeit over a wider range. This results in a nearly supersaturated state of calcium phosphate in body liquids driving mineral precipitation in soft tissues, which is actively prevented by calcification inhibitors. The hepatic plasma protein fetuin-A is a circulating mineralization inhibitor regulating calcium phosphate crystal growth and calcified matrix metabolism. Ectopic mineralization is associated with many pathological conditions aggravating their outcome. Current diagnostic methods lack sensitivity towards microcalcifications representing the initial stages of the process. Given the irreversibility of established calcifications, novel diagnostic tools capable of detecting nascent calcium phosphate deposits are highly desirable.

**Methods:** We designed fluorescent fusion proteins consisting of fetuin-A coupled to a green or red fluorescent protein derivate, mEmerald or mRuby3, respectively. The proteins were expressed in mammalian cell lines. Sequence optimization resolved folding issues and increased sensitivity of mineral binding. Chimeric proteins were tested for their ability to detect calcifications in cell cultures and tissue sections retrieved from calcification-prone mice.

**Results:** We employed novel genetically labeled fetuin-A-based fluorescent proteins for the detection of ectopic calcifications. We show that fetuin-A-based imaging agents are non-toxic and suitable for live imaging of microcalcifications beyond the detection limit of conventional staining techniques. The ability of fetuin-A to preferentially bind nascent calcium phosphate crystals allowed the resolution of histopathological detail of early kidney damage that went previously undetected. Endogenous expression of fetuin-A fluorescent fusion proteins allowed extended live imaging of calcifying cells with unprecedented sensitivity and specificity.

**Conclusion:** Ectopic microcalcifications represent a major clinical concern lacking effective diagnostic and treatment options. In this paper, we describe novel highly sensitive fetuin-A-based fluorescent probes for imaging microcalcifications. We show that fusion proteins consisting of a fetuin-A mineral binding moiety and a fluorescent protein are superior to the routine methods for detecting calcifications. They also surpass in continuous live cell imaging the chemically fluorescence labeled fetuin-A, which we established previously.

## Introduction

The ability of living organisms to incorporate calcium salts into their soft tissues has developed from a feature protecting vulnerable marine mollusks by shells and exoskeletons to the biomineralization of the skeleton in vertebrates. Biomineralization is physiologically restricted to a collagenous matrix of bones and teeth, and calcification is actively prevented in all other tissues by a heterogeneous group of local and circulating inhibitors of calcification 1. Various pathological conditions can compromise calcium homeostasis and lead to the formation of insoluble salts outside the skeletal system in a process known as ectopic calcification 2. In most cases, ectopic calcification is not harmful *per se* and develops as an adaptive reaction of the organism to delimit a pathological process, with examples being nodal calcifications in calcifying tendinitis, tuberculosis, lithopedion formation in missed abortions, and plaque-stabilizing macrocalcifications in atherosclerosis 3-5. In contrast, distinct calcification patterns, e.g., pro-inflammatory microcalcifications of atherosclerotic plaques, are considered detrimental as they aggravate the course of the underlying condition 4, 6. Current diagnostic methods lack sensitivity towards microcalcifications representing the initial stages of the process. A recent publication suggested the use of two-photon excitation (TPE) microscopy as a high-resolution non-destructive approach to visualizing the early stages of ectopic calcification 7. This method requires specific and sensitive fluorescent probes.

Dysregulated mineral metabolism, cell and tissue damage and lack of calcification inhibitors are prime causes of calcification. Proteins inhibiting calcification also called mineral chaperones avidly bind mineral and co-localize with established calcifications 8. The mineral chaperone fetuin-A is particularly interesting in this regard because it binds and stabilizes calcium phosphate precursor phases as colloids, thus allowing their transport and removal mitigating the risk of mineral precipitation 9. Accordingly, mice lacking the functional fetuin-A gene develop severe soft tissue mineralization starting in the lumen of microvasculature and have impaired bone formation 10-13. Previously we showed that fluorescent derivatives of the mineral-binding protein fetuin-A/α_2_-Heremans-Schmid glycoprotein (*Ahsg*) serve as sensitive and specific reagents to detect early-stage calcifications *ex vivo* and *in vivo* 13-16. We hypothesized that the mineral binding properties of fetuin-A might be improved by optimizing the protein-mineral binding interface. The calcium phosphate-binding of fetuin-A was originally attributed to negatively charged aspartic and glutamic acids in the amino-terminal cystatin-like domain 1 interacting with positively charged calcium-rich phases of hydroxyapatite-like calcium phosphates 17. Phosphorylated fetuin-A was shown to be preferentially included in calcium phosphate colloids 18. Recently, we determined the three-dimensional structure of the closest relative of fetuin-A, fetuin-B 19, 20. The fetuin-B structure allowed modeling of the complete structure of fetuin-A, which was later confirmed by AlphaFold2 21. This novel fetuin-A model suggested that the hitherto undefined intrinsically unfolded C-terminal region should also occupy an important role in protein-mineral binding apart from the amino-terminal cystatin-like domain identified earlier 9, 19, 22 because it harbors several more putative Ser/Thr phosphorylation sites. This is in line with the current understanding of the role of intrinsically disordered proteins in biomineralization 23. We figured that phosphomimetic versions of fetuin-A should further improve calcium phosphate mineral binding sensitivity and specificity.

Here we designed fluorescent fetuin-A-based fusion proteins and expressed them in mammalian cells. The mineral chaperone function of serum-derived and recombinant fetuins was tested using a calcium phosphate precipitation inhibition assay. Using fluorescent fetuin-A proteins as probes, we stained with high sensitivity calcified lesions on histological sections as well as in live calcifying cells. Endogenous expression of fluorescent fetuin-A fusion proteins in calcifying cells permitted the detection of calcification with previously unafforded low toxicity, sensitivity, and selectivity, constituting a novel calcification-reporting cell platform.

## Materials and methods

### Animals

All experiments involving animals were conducted in agreement with the recommendations of the Federation for Laboratory Animal Science Associations FELASA and were approved by the animal welfare committee of the Landesamt für Natur-, Umwelt- und Verbraucherschutz (LANUV) of the state of North Rhine Westphalia. All mice were maintained in environmental control cabinets on a 12-hour day/night cycle. Food and water were given *ad libitum*. Tissue samples were collected from 24-25 weeks old DBA/2-*Ahsg*^-/-^ mice. Animals were euthanized by an overdose of isoflurane prior to organ harvesting.

### Cell cultures

Primary human mesenchymal stromal cells (MSC) were obtained with written consent from patients undergoing total hip arthroplasty in the Clinic of Orthopedics, Trauma and Reconstructive Surgery of University Hospital Aachen UKA. Primary human vascular smooth muscle cells (VSMC) were isolated from healthy aortic tissue biopsies at Maastricht University Medical Center MUMC. Immortalized vascular smooth muscle cells (iVSMC) were derived from primary commercial cell lines. All human cells were isolated in accordance with local, state, and federal laws and regulations. Human tissue banking was approved by ethical review boards for research and diagnostic procedures at UKA (EK 300/13) or MUMC. Collection, storage and use of tissue and patient data were performed in agreement with the Dutch Code for Proper Secondary Use of Human Tissue (https://www.federa.org/codes-conduct accessed on 30 July 2021). All parts of this study complied with the Declaration of Helsinki. Human embryonic kidney cell line HEK293 was from ATCC.

Osteogenic, mineralization-competent “mineralizing” cells (MSC) and non-osteogenic mineralization-incompetent “calcifying” cells (VSMC, iVSMC, HEK293) were treated with either osteogenic medium OM (MSC) or with calcifying media (VSMC, iVSMC, HEK293).

### Human mesenchymal stromal cells

Human mesenchymal stromal cell harvest and culture followed published protocols 24, 25. After isolation, MSC were maintained in Mesenpan medium containing 2% fetal bovine serum (FBS), 1% ITS-plus (insulin, transferrin, selenic and linoleic acids, bovine serum albumin), 1 nM dexamethasone, 100 µM ascorbic-acid-2-phosphate, 10 ng/mL epidermal growth factor (all from Pan-Biotech, Germany), 80 U/mL penicillin, 80 µg/mL streptomycin, and 1.6 mM L-glutamine (all from Gibco, Germany). Culture medium was exchanged every 3-4 days. Following expansion and characterization by flow cytometry, the cells were seeded on 8-well Ibidi µslides (Ibidi GmbH, Germany) at a density of 1x10^4^ cells/cm^2^ and cultured for 72 h in low-glucose Dulbecco's Modified Eagle's Medium (DMEM, glucose content 1 g/L) (Thermo Scientific, Germany) supplemented with 10% FBS, 100 U/mL penicillin, 100 µg/mL streptomycin, and 2 mM L-glutamine (base medium). To trigger osteoblastic differentiation medium was switched to osteogenic medium, consisting of the above DMEM also containing 100 µM dexamethasone, 10 mM β-glycerophosphate, and 0.05 mM L-ascorbic acid-2-phosphate (all from Sigma Aldrich, Germany). OM was exchanged twice a week.

### Primary human vascular smooth muscle cells

Primary human VSMC isolated from surgical biopsies of non-atherosclerotic thoracic aortas 15 were cultured in DMEM supplemented with 20% FBS, 100 U/mL penicillin, 100 µg/mL streptomycin, and 2 mM L-glutamine at 37°C and 5% CO_2_. Cells between passages 5 and 9 were seeded into a 48-well plate at a seeding density of 1x10^4^ cells/cm^2^. To trigger calcification, medium was switched to DMEM, 0.5% FBS, and 3.6 mM total calcium. The reduced serum content in culture medium increased cell calcification as serum contains proteins and small molecules inhibiting calcification. Culture medium was not exchanged during the experiment.

### Immortalized human vascular smooth muscle cells

Human vascular smooth muscle cells iVSMC were immortalized by transduction using the SV40LT and HTERT systems. Clone IM1 was isolated and maintained at 37 °C and 5% CO_2_ in M199 medium (Thermo Scientific), 10% FBS, 100 U/mL penicillin, 100 µg/mL streptomycin, and 2 mM L-glutamine. For confocal microscopy, iVSMC were seeded on 8-well Ibidi µslides (80826, Ibidi GmbH, Gräfelfing, Germany) at a density of 2x10^4^ cells/cm^2^ and attached for 24 hours. To trigger calcification, medium was switched to M199 base medium as above adjusted to 4.3 mM total calcium and 3.0 mM total phosphate. Cells were cultured for five days with one medium exchange.

### Human embryonic kidney cells

To mimic kidney calcification, human embryonic kidney 293 cells (HEK293) (CRL-1573, ATCC, Manassas, USA) were employed. Cells were maintained in DMEM supplemented with 2% FBS, 100 U/mL penicillin, 100 µg/mL streptomycin, and 2 mM L-glutamine for 3 passages prior to experiments. For procalcifying conditions, culture medium was adjusted to 4.3 mM total calcium and 3.0 mM total phosphate at 65-70% confluency. HEK293 line is an established cell platform for large-scale protein expression. To generate cells that would simultaneously calcify and produce a calcification-reporting marker, a TOPO3.4 vector encoding *mFA-mRuby3* gene was introduced into the cells 4 h prior to calcification induction using the TransIT-293™ (2700, Mirus Bio, Madison, USA) transfection reagent according to the manufacturer's instructions. To enhance the protein expression, the culture medium was supplemented with a 1% non-essential amino acids solution (11140050, Life Technologies, Grand Island, USA). To access calcification, live fluorescence imaging was performed on day 3 post-transfection. Culture medium was exchanged shortly before microscopy to decrease background fluorescence caused by the fusion proteins released in the medium.

### Purification and fluorescence labeling of bovine fetuin-A

Serum-derived bovine fetuin (F2379, Sigma, Taufkirchen, Germany) was subjected to size-exclusion chromatography and fractions containing the monomeric protein were collected as described 14. The purity of the obtained fractions was confirmed by SDS-PAGE. Bovine fetuin-A was labeled with Alexa Fluor™ 488 NHS ester (Thermo Scientific) according to the manufacturer's protocol and aliquoted into screw cap vials. The labeled protein was frozen in liquid nitrogen and stored at -20°C.

### Design and expression of recombinant fetuin-A proteins

Full-length mouse fetuin-A mFA (UniProtKB entry P29699) was chosen as the reference protein for functional assays and for the design of fusion proteins and variants thereof. All recombinant fetuin-A vectors were ordered as synthetic genes from Life Technologies GmbH (Darmstadt, Germany).

Fluorescent fusion proteins mFA-mEmerald and mFA-mRuby3 comprised full-length murine fetuin-A connected to photostable monomeric green fluorescent protein mEmerald and red fluorescent protein mRuby3, respectively 26. Both fusion proteins contained the sequence GGGS as a flexible linker between mFA and fluorescent protein, and the C-terminal 6-His tag for purification. Proteins were expressed in the suspension-adapted Chinese hamster ovary cell line (ExpiCHO-S™/CHO) (Thermo Scientific, Waltham, USA). Several candidate polypeptide sequences with varying signal peptides, linker regions and purification tags were designed and tested using the ProtParam tool of the Expasy server 27. Sequences with the lowest estimated instability index were selected for downstream processing. Next, reverse translation was used to generate nucleotide sequences for the respective proteins. To further improve gene expression, codon optimization toward the mammalian host cell line was performed. The Kozak consensus sequence and the HindIII restriction site were introduced upstream of the N-terminus, and the resulting constructs were cloned into the pcDNA™ 3.4 TOPO mammalian expression vector. Plasmids were obtained from Life Technologies GmbH (Darmstadt, Germany) and propagated in the endonuclease I-deficient NEB Turbo *E. coli* strain and purified using the PureYield™ Plasmid Midiprep System (Promega, Madison, USA). The transfection-grade DNA was introduced into the CHO cells by lipofection. Cells were imaged daily to detect fluorescent protein. Histidine-tagged recombinant proteins were harvested on day 8 post-transfection and recovered by immobilized metal affinity chromatography (IMAC). Imidazole-containing elution buffer was exchanged to the HEPES-buffered saline using Zeba™ spin desalting columns (89894, Thermo Scientific, Rockford, USA) and the elution fractions were analysed by SDS-PAGE. Immunodetection of expressed proteins was performed by western blotting using either rabbit anti-mouse fetuin-A antiserum produced in-house or the HisProbe™-HRP nickel-activated conjugate (15165, Thermo Scientific, Waltham, USA).

### Phosphomimetic fetuin-A proteins

We replaced serine or threonine residues at positions 135, 138, 305, 309, 312 and 314 of mFA with glutamic acid to mimic the full phosphorylation of residues listed in UniProt P29699 as putative phosphorylation sites. The prefix “pm-“ denotes the respective phosphomimetic fetuin-A variants in this study, i.e. pm-mFA, pm-mFA-mEmerald, and pm-mFA-mRuby3.

### Functional assessment of recombinant fetuin-A proteins

To test the activity of expressed fetuin-A proteins, a calcium phosphate precipitation inhibition assay was used. The assay interrogates the functional property of fetuin-A to form mineral-protein complexes in solutions supersaturated with respect to calcium and phosphate, preventing the growth of crystallization nuclei. Proteins were added from 0 - 8 µM into assay buffer (50 mM Tris, 140 mM NaCl pH 7.4 at 37 °C). All stock salt solutions were sterile filtered (pore diameter ≤ 0.2 µm). Phosphate and calcium were added to 3 mM Na_2_HPO_4_/NaH_2_PO_4_ and 5 mM CaCl_2,_ respectively, in a total volume of 200 µL assay buffer. After each addition, the solution was thoroughly mixed to avoid local supersaturation, and, until incubation, all mixtures were stored on ice (4°C). After 1 h of incubation at 37°C, centrifugation (10 min, 20,000 x g, 4°C) was performed to separate the supernatant from the precipitate. First, 50 µL of 0.6 M HCl and then 50 µL of ammonium buffer (45 mM NH_4_Cl pH 10.5, 5% v/v NH_4_OH) were stepwise added onto half of the supernatant (100 µL) and mixed thoroughly. Similarly, to the remaining 100 µL supernatant including pellet, 50 µL 0.6 M HCl and 50µL ammonium buffer (45 mM NH_4_Cl pH 10.5, 5% v/v NH_4_OH) were successively added and mixed thoroughly to dissolve the pellet. Calcium content in the supernatant and in the corresponding precipitate was determined photometrically using Randox Calcium Reagent™ (Randox Laboratories, Crumlin, UK) according to the manufacturer's instructions.

### Toxicity testing of fluorescent fetuin-A proteins

To assess toxicity, we exposed MSC to 12 µM fluorescent fetuin-A proteins corresponding to twice the amount used for 2-hour short live cell staining and 50-fold the amount used for one-week extended live staining with bFA-Alexa 488, mFA-mEmerald and mFA-mRuby3. The cells were seeded in 24-well plates at a density of 5x10^3^ cells/cm^2^ and maintained in low-glucose DMEM, 10% FBS. After 1 week of culture, fresh medium was exchanged and 12 µM fluorescent fetuin-A protein was added for two hours. Cells were washed and incubated for another 3 days. Cells were detached by trypsinization and viability was assessed by trypan blue dye exclusion. Cell viability was calculated as the ratio of unstained cells to the total number of cells counted using a hemacytometer.

### Cell staining and microscopy

To identify calcified areas in MSC and iVSMC by short-term live staining, fluorescent proteins or fluorescein derivative calcein (Sigma Aldrich, Germany) were added to the cells at a final concentration of 6 µM or 0.2 mM, respectively. Cells were stained for 1.5 h at 37 °C and 5% CO_2_. Unbound protein was rinsed with phosphate-buffered saline (PBS). Cells were counterstained using 1 µg/mL Hoechst 33342 and either 5 µg/mL CellTracker™ Green CMFDA or 10 µg/mL CellTracker™ CM-DiI. After 15 min incubation at 37 °C, cells were visualized with a Zeiss LSM 710 confocal laser scanning microscope. After recording fluorescence micrographs, cells were rinsed, fixed with 4% paraformaldehyde and post-stained with Alizarin Red S 28. For extended live staining of calcifying VSMC, the culture medium was supplemented with 0.24 µM (20 µg/mL) of mFA-mRuby3 as described 15. VSMC image acquisition was performed using a Cytation 3 automated cell imaging system (BioSPX, Abcoude, The Netherlands) on days 1, 3 and 7. Image analysis was performed using ImageJ software (v. 1.53a) 29. Quantification of calcified areas in HEK293 cells and co-localization analyses were carried out using Fiji software (v. 2.1.0/1.53c) 30.

### Histology

For histologic examination, organs of euthanized mice were harvested through a midline laparotomy and rinsed with PBS. Hearts were additionally perfused with ice-cold PBS to remove blood. The samples were then frozen in the Tissue-Tek™ compound and 5-µm cryosections were made. Tissue sections were placed on glass slides and circled with a paraffin pen. Sections were incubated with 1µM fluorescent protein solution (~50 µg/mL, bFA-AF488; ~85 µg/mL, pm-mFA-mEmerald and mFA-mRuby3, respectively) for 4 h at 37°C. Sections were rinsed with PBS three times. Fluorescence images were recorded using a Leica DMI 6000 microscope. The fluorescent probe was removed by rinsing the slides in phosphate-buffered saline supplemented with 0.05% Tween 20™. Post-staining was carried out using Alizarin Red S (5% in water adjusted to pH 4.2 with HCl).

### Statistics

Statistical analysis was performed using GraphPad Prism 9. Protein toxicity was analyzed on triplicates using one-way ANOVA. Fluorescence of calcifying VSMC was analyzed on five replicates each using one-way ANOVA with Tukey correction for multiple comparisons.

## Results

### Analysis of fluorescent fusion proteins

**Figure 1:**
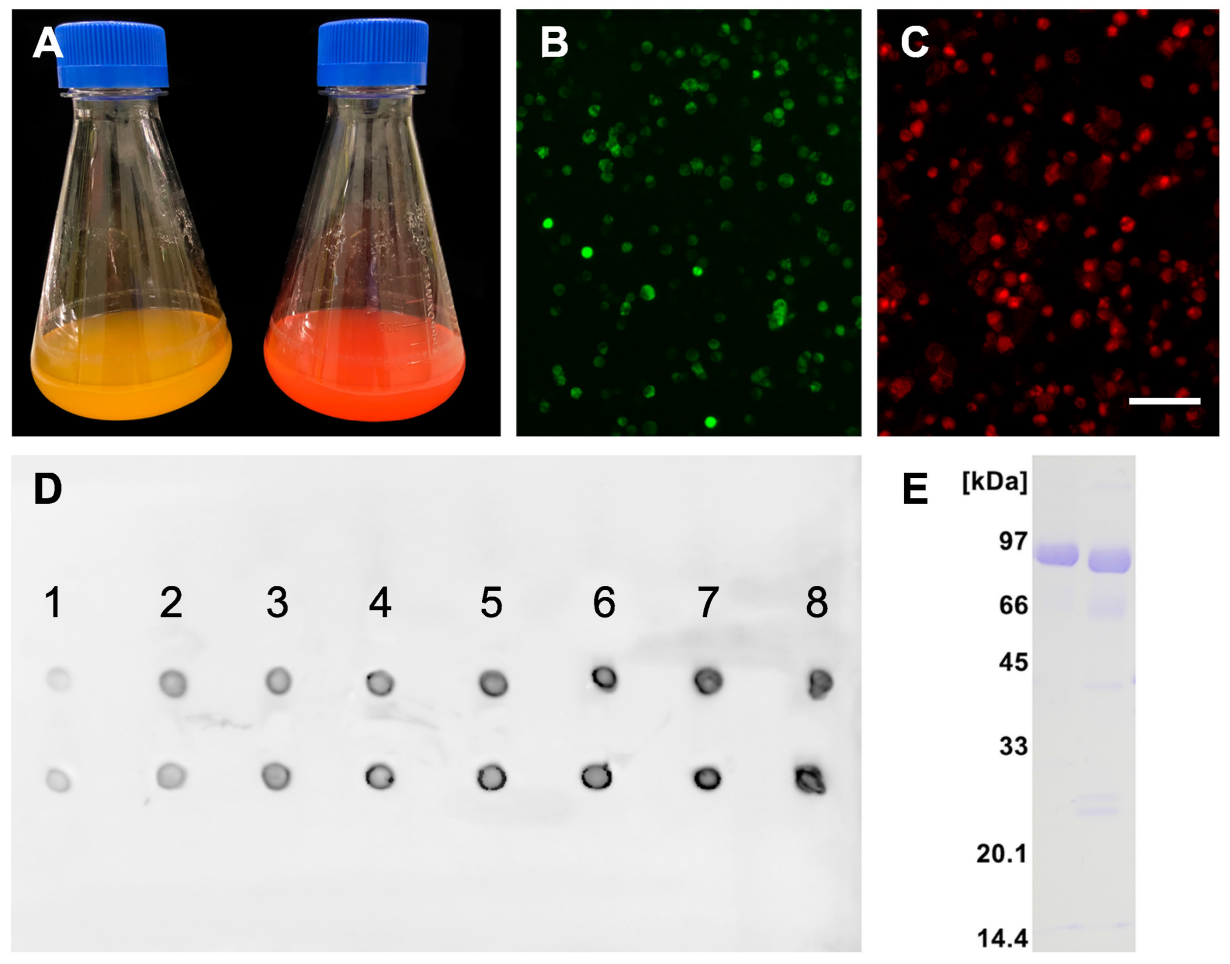
Recombinant fluorescent fetuin-A expression. **(A)** Flasks containing CHO cells expressing mFA-mEmerald (left) and mFA-mRuby3 (right); **(B, C)** Representative fluorescence micrographs of protein-expressing cells at day 3 post-transfection with mFA-mEmerald (B) and mFA-mRuby3 (C). Scale bar 75 µm. **(D)** Time course of recombinant mFA-mEmerald (top row) and mFA-mRuby3 (bottom row) expression. Numbers indicate the day post-transfection. Dots represent 1.5 µL aliquots of CHO cell culture supernatant sampled on days 1-8 post-transfection. Recombinant proteins were detected with a HisProbe-HRP conjugate. **(E)** SDS-PAGE of recombinant mFA-mEmerald (left lane) and mFA-mRuby3 (right lane) after His-trap chromatography showing high yield, high purity, and the expected molecular weights of the recombinant fusion proteins.

Recombinant protein production was carried out following a high titer protocol in suspension-adapted CHO cells. Figure 1A shows typical macroscopic views of cell cultures expressing fluorescent mFA-mEmerald (left) and mFA-mRuby3 (right) on day 8 after transfection. Figures 1B, C show fluorescence micrographs of the respective cells on day 3 post-transfection. Comparison with the total number of cells visible in brightfield (not shown) suggested that 19% and 31% of the cells expressed mFA-mEmerald (B) and mFA-mRuby3 (C), respectively. We chose day 8 for harvesting medium because the secreted recombinant protein in the medium peaked on that day as estimated using spot blotting (Figure 1D). After medium collection, proteins were purified by IMAC, desalted by size exclusion chromatography, and analyzed by SDS-PAGE. Figure 1E shows Coomassie-stained fusion proteins migrating as single bands at the expected molecular size of ~85 kDa for mFA-mEmerald (left lane) and mFA-mRuby3 (right lane), respectively.

Fetuin-A is a major constituent of fetal bovine serum and therefore, non-toxic by default. Large amounts of recombinant fetuin-A were expressed by CHO cells confirming the non-toxicity of fetuin-A. To test if chemically or genetically labeled forms of fetuin-A retained their cytocompatibility, we incubated MSC with fluorescent fetuin-A proteins for 2 h at a concentration twice higher than used for short-term and 50-fold higher than used for long-term live calcification imaging in later experiments. Cell viability measurements performed at 72 h post-incubation with bovine fetuin-A chemically labeled with AlexaFluor 488 (bFA-AF488), recombinant murine fetuin-A fused with mEmerald (mFA-mEmerald) or mRuby3 (mFA-mRuby3) showed similarly high cell viability like PBS treated cells (Figure S1). Collectively, these findings confirmed that labeled fetuin-A was suitable for live cell and tissue imaging.

**Figure 2:**
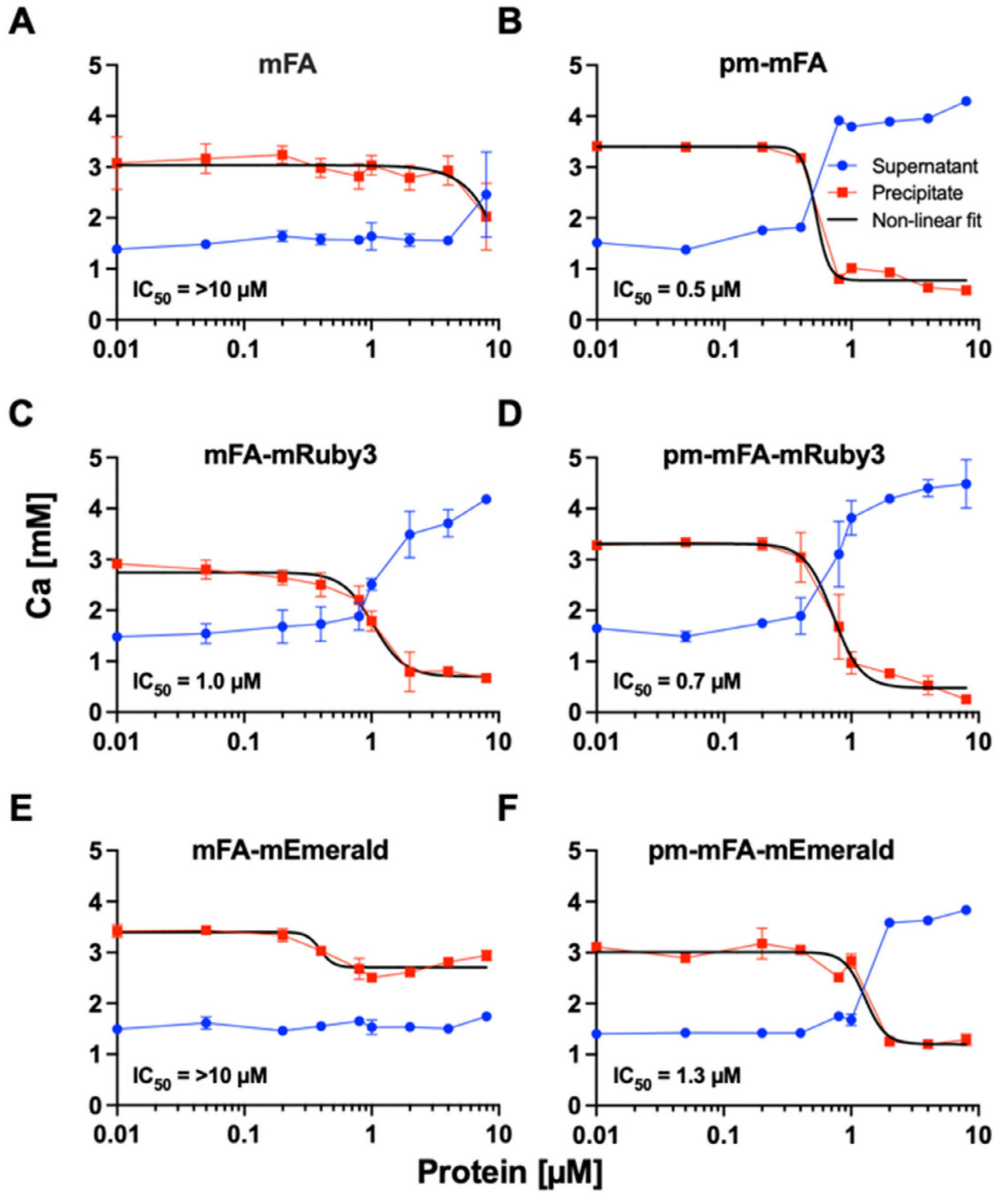
Precipitation inhibition assay. Blue dots represent calcium in the supernatant, red squares show calcium in the precipitate. **(A)** Recombinant carboxy-terminally His-tagged mFA started to inhibit calcium phosphate precipitation at ~10 µM. **(B)** Replacement of putative phosphorylation sites with glutamic acid in pm-mFA enhanced inhibition to IC_50_ 0.5 µM. **(C)** mFA-mRuby3 fusion protein inhibited precipitation of calcium phosphate with IC_50_ 1 µM. **(D)** pm-mFA-mRuby3 had an IC_50_ value of 0.7 µM. **(E)** mFA-mEmerald showed little inhibition of calcium phosphate precipitation. **(F)** pm-mFA-mEmerald inhibited effectively with IC_50_ 1.3 µM. Individual data points represent mean ± SD.

Next, we tested if recombinant mFA (fusion) proteins were functionally intact in terms of calcium phosphate binding. To this end, we performed precipitation inhibition assays with increasing amounts of protein added to a test solution supersaturated with respect to calcium and phosphate. In this test, calcification inhibitors like mFA prevent the growth of crystal nuclei and thus the formation of a precipitate. Recombinant carboxy-terminally His-tagged mFA only started to inhibit calcium phosphate precipitation at ~10 µM (Figure 2A). Replacing serine or threonine residues at positions 135, 138, 305, 309, 312 and 314 with glutamic acid to mimic full constitutive phosphorylation of mFA residues listed in UniProt P29699 enhanced inhibition to an IC_50_ value of 0.5 µM suggesting that fetuin-A phosphorylation regulates mineral binding (Figure 2B).

Figures 2C, D illustrate that the fusion protein mFA-mRuby3 prevented nascent calcium phosphate precipitation with an IC_50_ value of 1 µM and that the respective phosphomimetic version pm-mFA-mRuby3 had an even lower IC_50_ value of 0.7 µM. mFA-mEmerald like mFA did not inhibit calcium phosphate precipitation at concentrations up to 10 µM (Figure 2E), while the phosphomimetic version pm-mFA-mEmerald inhibited well with an IC_50_ value of 1.3 µM (Figure 2F). Collectively, these results show that replacing putative Ser/Thr phosphorylation sites with the acidic amino acid Glu enhanced calcium phosphate precipitation inhibition and thus mineral interaction at least 10-fold.

### Imaging of cell-mediated mineralization and calcification

**Figure 3:**
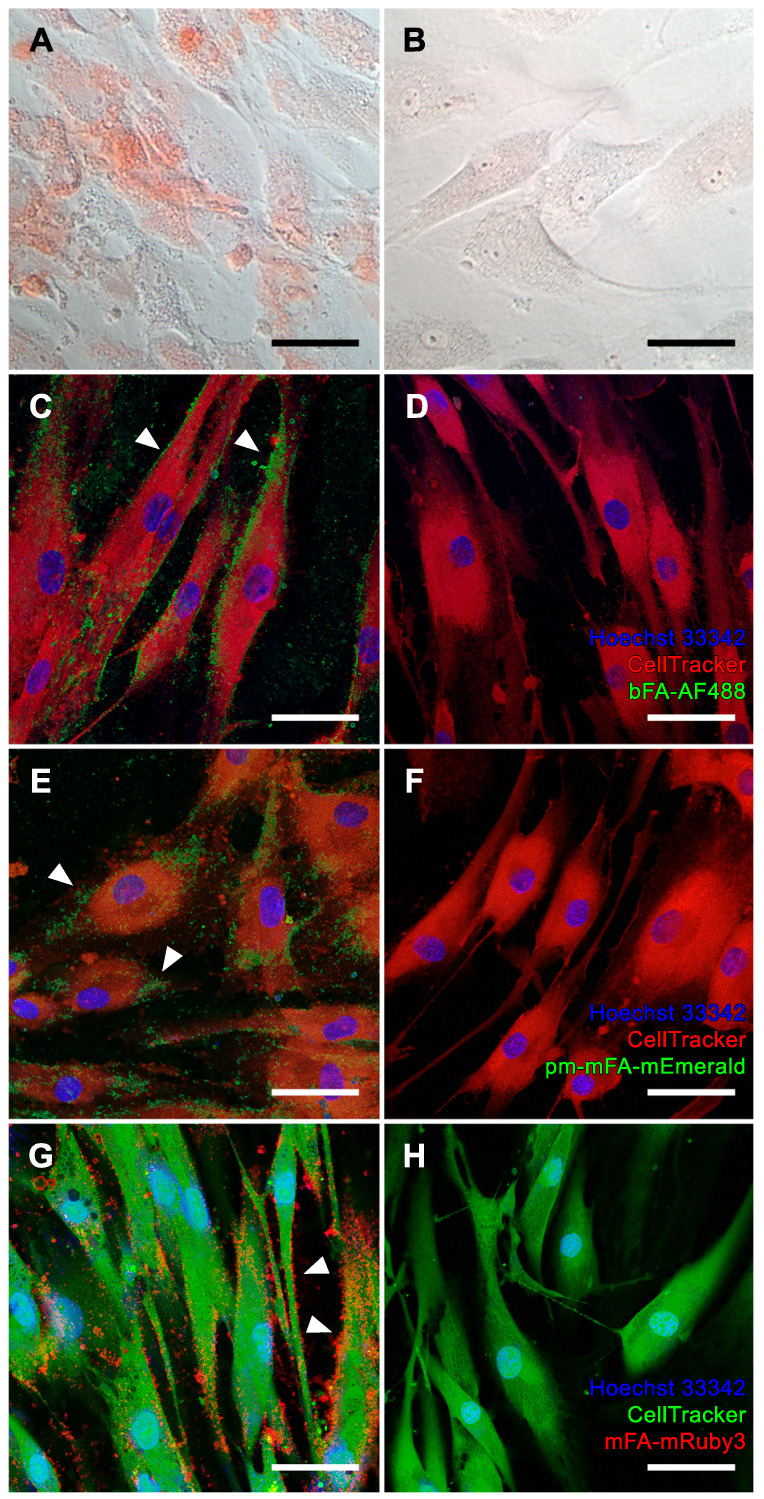
Mineralization of MSC. **(A, B)** Brightfield microscopy, Alizarin Red S staining. **(A, C, E, G)** MSC cultured in osteogenic medium. **(B, D, F, H)** MSC maintained in basal medium. **(C-H)** Confocal laser scanning microscopy of cells stained with **(C, D)** bFA-AF488 (green), **(E, F)** pm-mFA-mEmerald (green) or **(G, H)** mFA-mRuby3 (red). MSC cultured in osteogenic medium show sub-micrometer mineralization of the extracellular matrix demarcating cell outlines (white arrowheads) while cells cultured in basal medium remain unstained after incubation with fluorescent fetuin-A probes. Nuclei were stained with Hoechst 33342 and cell bodies were stained with CellTracker™ CM-DiI **(C-F, red)** or CellTracker™ CMFDA **(G-H, green)**. Scale bars 50 µm.

We chose osteogenic differentiation of human bone-derived MSC as a model of physiological bone mineralization. MSC differentiated in osteogenic medium (OM) for two weeks stained positive with Alizarin Red S, indicating matrix mineralization (Figure 3A), while MSC cultured in non-osteogenic DMEM did not stain Alizarin Red S positive (Figure 3B). Next, we stained live OM-differentiated MSC stained with bFA-AF488 or with fluorescent pm-mFA-mEmerald and mFA-mRuby3 and imaged the cells at high resolution using confocal laser microscopy. In each case, sub-micrometer-sized mineralization foci demarcating the cell outline (white arrowheads) indicate osteoblastic extracellular matrix mineralization (Figures 3C, E, G). These fine features were not resolved using conventional Alizarin Red S staining. The absence of fetuin-A-derived fluorescence in non-mineralized MSC kept in basal DMEM confirmed the high selectivity of fetuin-A-based mineral staining (Figure 3D, F, H).

**Figure 4:**
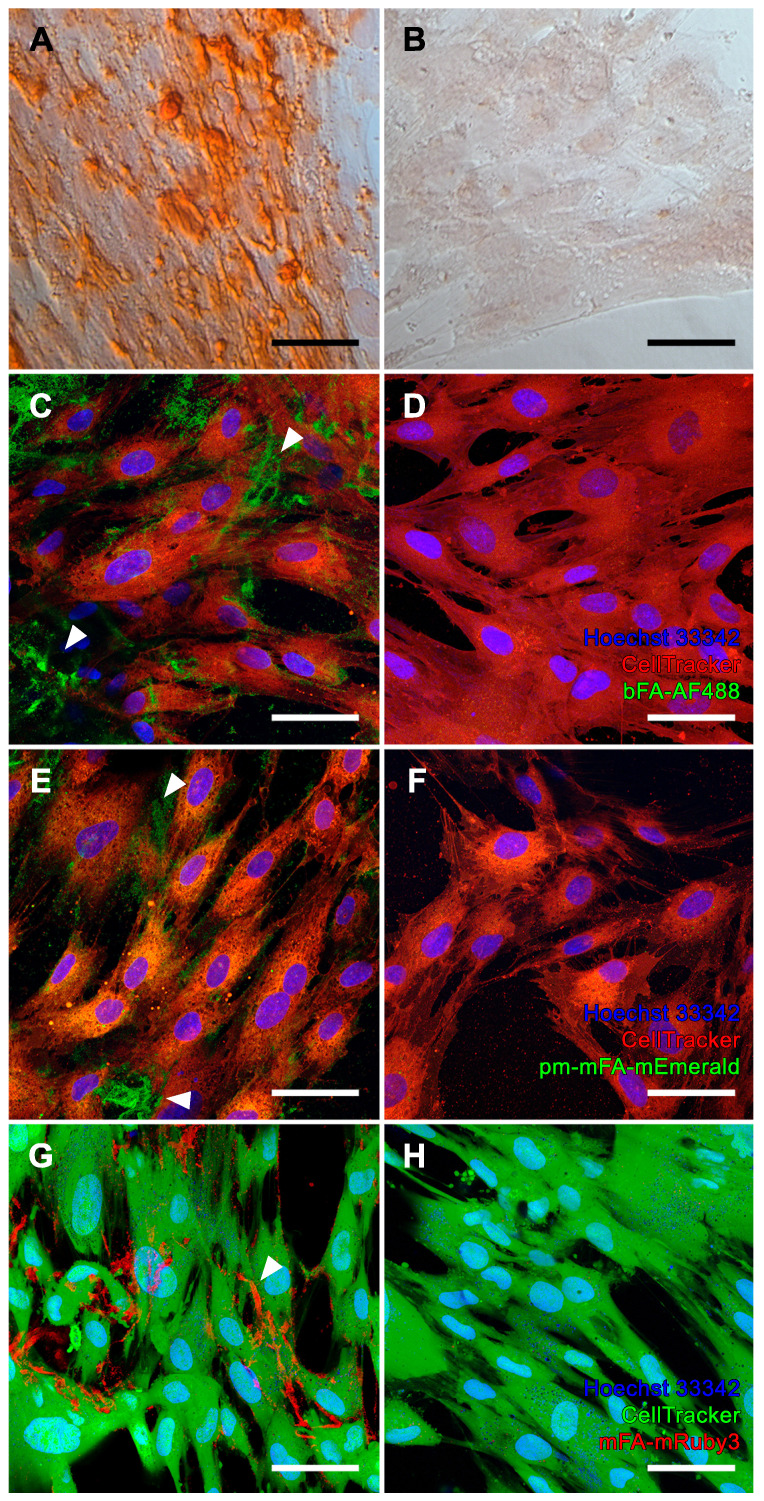
Calcification of iVSMC. **(A, B)** Brightfield microscopy, Alizarin Red S staining. **(A, C, E, G)** iVSMC cultured in calcification medium. **(B, D, F, H)** iVSMC maintained in basal medium. **(C-H)** Confocal laser scanning microscopy of cells stained with **(C, D)** bFA-AF488 (green), **(E, F)** pm-mFA-mEmerald (green) or **(G, H)** mFA-mRuby3 (red). iVSMC cultured in calcification medium show strongly stained patchy areas demarcating the irregular patterns of dystrophic iVSMC calcification (white arrowheads) while cells cultured in basal medium remained unstained after incubation with fluorescent fetuin-A probes. Nuclei were stained with Hoechst 33342 and cell bodies were stained with CellTracker™ CM-DiI **(C-F, red)** or CellTracker™ CMFDA **(G-H, green)**. Scale bars 50 µm.

We chose iVSMC treated with calcification medium as a model of pathological vascular calcification. We kept iVSMC in calcification medium for five days. The cells stained positive with Alizarin Red S indicating calcification (Figure 4A). Unlike osteogenic MSC, which mineralized their entire cell outline (Figure 3A), iVSMC had patchy staining of cells and agglomerated cell debris, indicating dystrophic calcification. iVSMC cultured in M199 basal medium lacked Alizarin Red S positive staining (Figure 4B). Next, we performed live fluorescence staining of iVSMC using bFA-AF488 (Figures 4C, D) or pm-mFA-mEmerald (Figures 4E, F) or mFA-mRuby3 (Figures 4G, H). Like the Alizarin Red staining, high-resolution fluorescence microscopy revealed strongly stained patches demarcating the irregular patterns of dystrophic iVSMC calcification (Figures 4C, E, G). Again, the high selectivity of mineral staining was validated by the absence of any fetuin-A-derived fluorescence signal in non-calcified iVSMC kept in basal M199 (Figures 4D, F, H). All cell cultures had fine, granular intercellular fluorescence representing mineral precipitated on the culture plates (Figures 3C, E; Figures 4C, E, G), a feature of all cell-based mineralization assays. These fine granular precipitates were however readily distinguishable from the cell membrane associated mineralized ECM in Figure 3 and the calcified cell remnants in Figure 4, which are demarcated by arrowheads. We then asked whether corresponding staining patterns in MSC and iVSMC can be observed when using an established fluorescent calcium dye. To this end, we performed simultaneous staining of the mineralizing and calcifying cells with mFA-mRuby3 and the fluorescent mineral stain, calcein. Similar to Alizarin Red S dye, calcein predominantly detected larger features present in MSC and iVSMC, leaving most of the MSC-associated granular calcifications unstained (Figure S2, left panel). This observation was further supported by co-localization analysis of two fluorescent channels that revealed a partial overlap between mFA-mRuby3 and calcein signals in MSC (Figure S2, upper right plot, PCC = 0.42), but a strong overlap in iVSMC culture (Figure S2, lower right plot, PCC = 0.90).

### Histological staining

**Figure 5:**
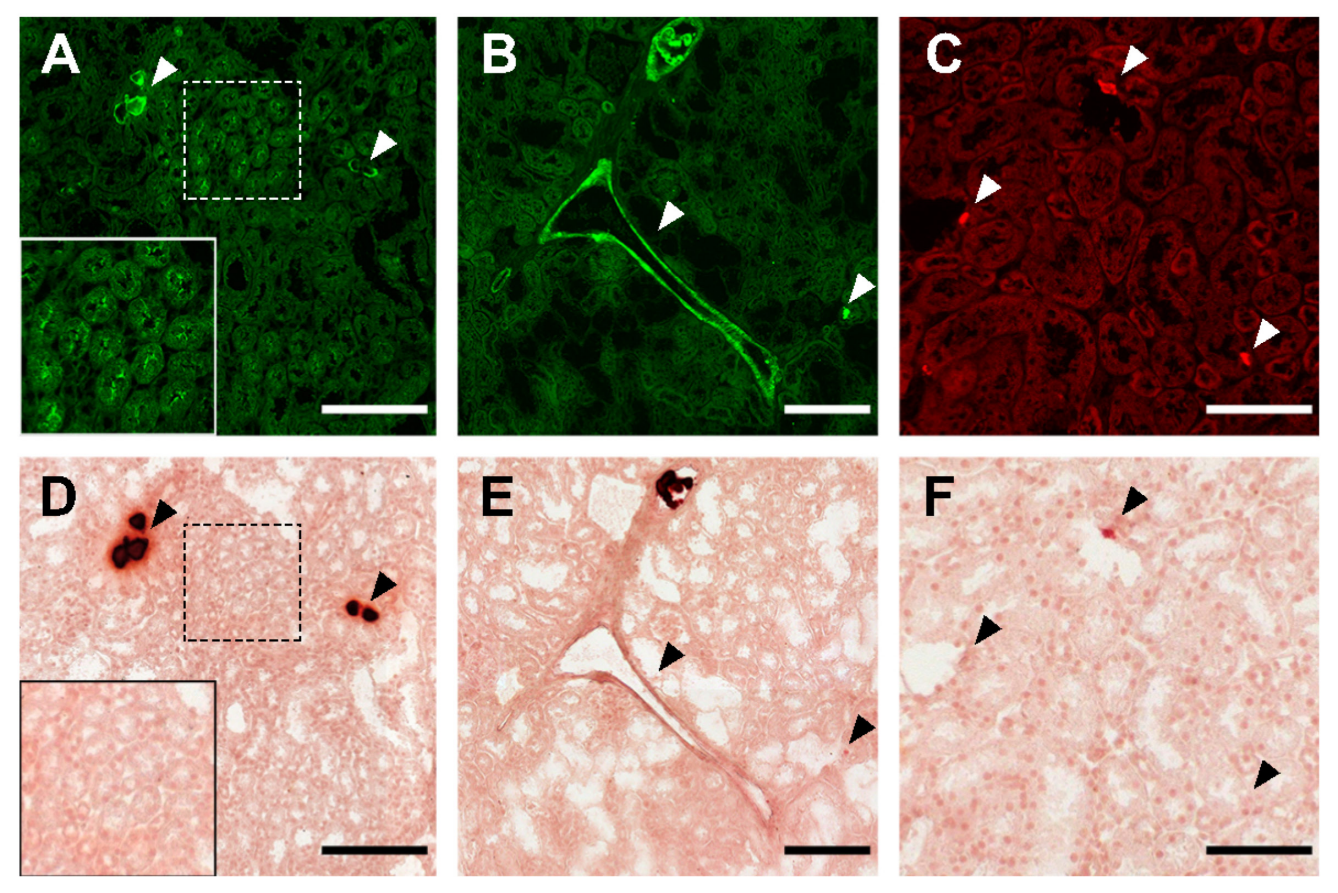
Kidney calcification staining. **(A)** Large and small calcified lesions (white arrowheads), and area of tubular calcification (white box) stained with bFA-AF488. **(B)** Calcified tunica media, large and small lesions stained with pm-mFA-mEmerald. **(C)** Small calcified lesions stained with mFA-mRuby3 (white arrowheads). **(D-F)** Post-staining with Alizarin Red S of the identical sections shown in **A-C**. Black arrowheads copy the positions of white arrowheads in **A-C**. Scale bars 200 µm **(A, B, D, E)** and 100 µm **(C, F)**.

Having established that fluorescent fetuin-A stained mineralized MSC and calcified iVSMC, we asked if calcified tissue sections could also be stained. We stained kidney sections from fetuin-A-deficient mice on a DBA/2 genetic background representing one of the most robust models of soft tissue mineralization. These animals develop dystrophic calcification in nearly all soft tissues by the age of 12 weeks. Fluorescent fetuin-A fusion proteins pm-mFA-mEmerald and mFA-mRuby3 strongly decorated large and small calcified lesions marked by arrowheads (Figures 5A-C) and kidney tubule calcification boxed in Figures 5A, D. Unlike fluorescent fetuin-A staining, Alizarin Red S post-staining of the identical sections (Figure 5D-F) only detected large lesions, but not small lesions (Figure 5C) and the tubular calcification (Figure 5A). Similarly, simultaneous staining of a calcified kidney section with calcein and fetuin-A-mRuby3 showed the inability of calcein to detect subtle calcifications, whereas larger calcifications were clearly discernible (Figure S3, lower panel).

### Continuous live staining of calcifying cells

**Figure 6:**
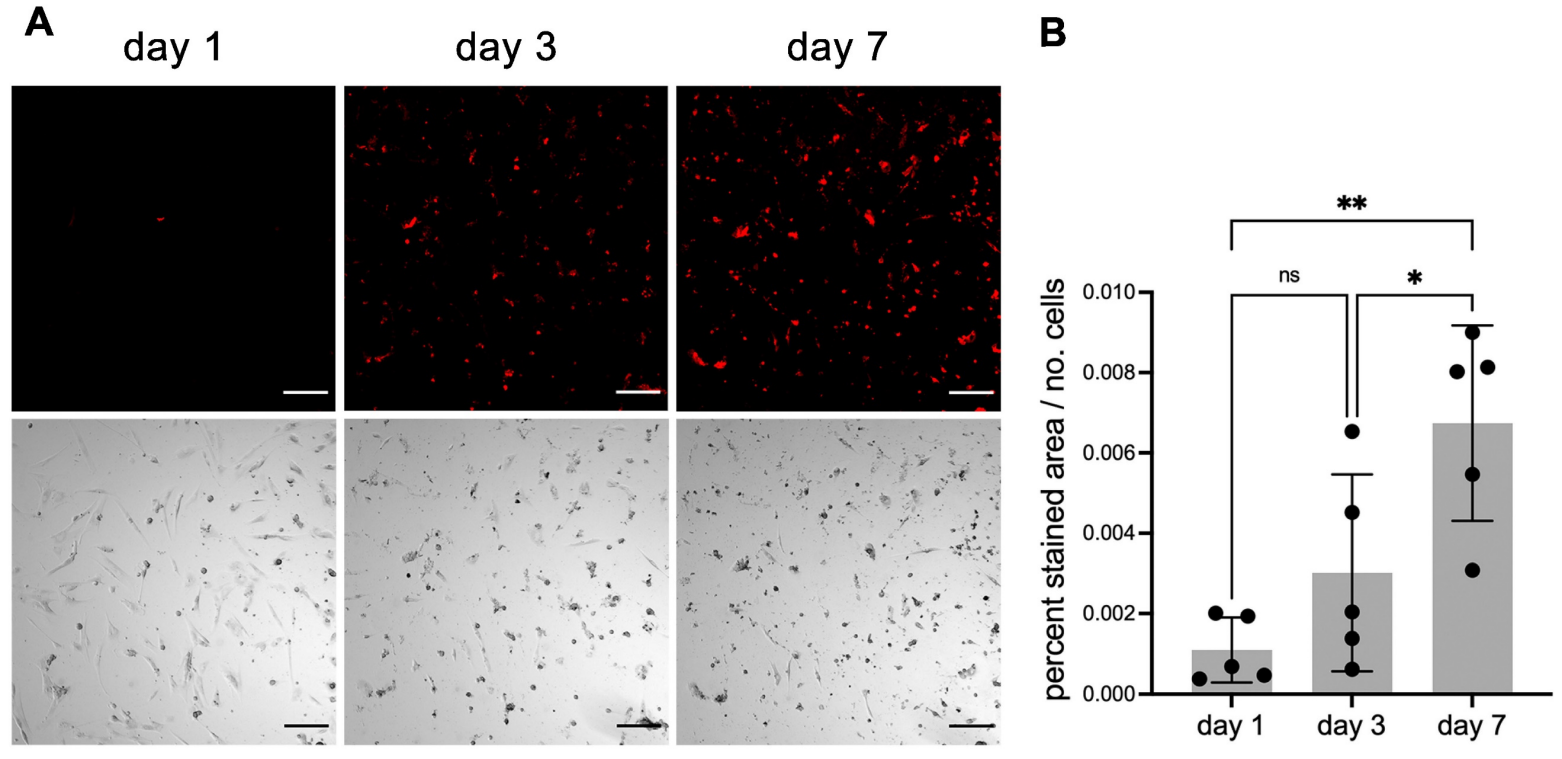
Continuous live staining of calcifying cells. VSMC were cultured in calcification medium also containing 20 µg/mL mFA-mRuby3. **(A)** Fluorescence (upper panel) and brightfield images (lower panel) of calcifying VSMC. Scale bars 200 µm. **(B)** Fluorescence of calcifying VSMC. Data represent the ratio of fluorescence area (%) divided by the number of cells (ns, not significant; * < 0.05 P-value, ** < 0.01 P-value). Mean ± SD. n = 5.

Next, we tested if fluorescent fetuin-A probes would permit continuous live staining of calcifying cells. Primary human VSMC were cultured for 1 week in calcification medium containing 20 µg/mL mFA-mRuby3. This medium was optimized for low cell proliferation and moderate calcification propensity to allow the continued longitudinal study of calcification in viable cells. Fluorescence was normalized to the number of cells recorded on days 1, 3 and 7. Fluorescence imaging revealed a steady increase in the number of cells stained with mFA-mRuby3 (Figure 6A). This observation was confirmed by a quantitative assessment of fluorescence signal normalized to the number of cells (Figure 6B).

### Endogenous continuous live staining of calcifying cells

**Figure 7:**
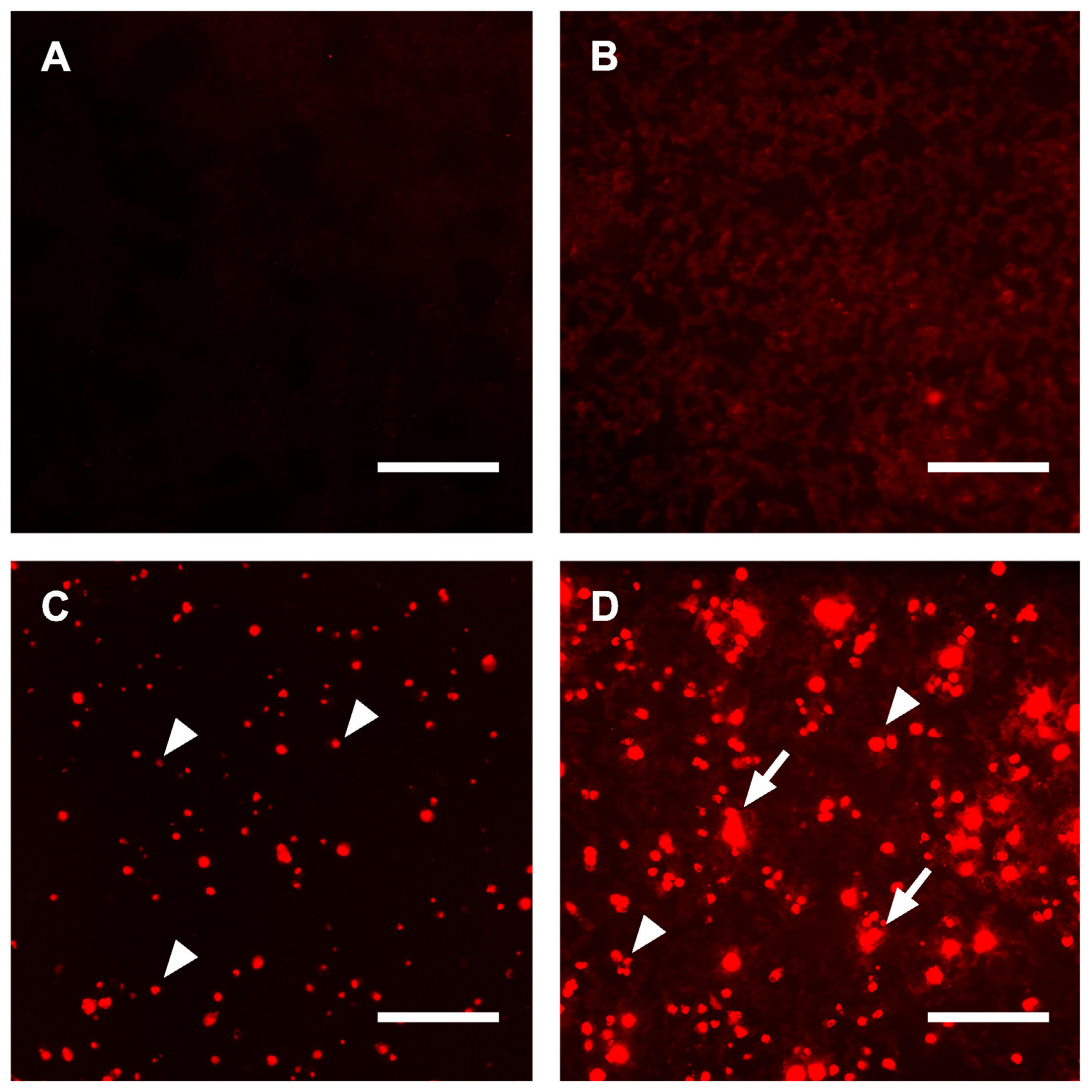
Calcifications in HEK293 cells. **(A)** Untransfected HEK293 cells cultured in DMEM and post-stained for 1 day using culture supernatant from mFA-mRuby3-transfected cells. **(B)** Untransfected HEK293 cells cultured in calcification medium showed dim fluorescence following post-staining for 1 day with culture supernatant from mFA-mRuby3-transfected cells,** (C)** mFA-mRuby3-transfected HEK293 cells cultured in basal DMEM present as bright red dots (arrowheads) on day 3 post-transfection. **(D)** mFA-mRuby3-transfected HEK293 cells cultured in calcification medium present as bright red dots (arrowheads) on day 3 post-transfection. In addition, large fluorescent lesions demarcate calcified cells (arrows). Scale bars depict 150 µm.

Staining of tissue sections and viable cells with chemically or genetically labeled fetuin-A variants was successfully used for high-resolution microscopy and live staining. Next, we asked whether endogenous expression of fluorescent fetuin-A would increase the signal intensity of calcifying cells, thus constituting a high-sensitivity reporter cell platform for calcification studies. For traditional staining, we cultured untransfected HEK293 cells in DMEM under normal conditions and stained the cells using culture supernatant from mFA-mRuby3-transfected HEK293 cells for 1 day. Figure 7A shows the lack of fluorescence in these cells. In contrast, untransfected HEK293 cells cultured in calcification medium and stained with culture supernatant from mFA-mRuby3-transfected HEK cells showed dim fluorescence like the background staining seen in Figure 5C and Figure 6B. Next, we transfected HEK293 cells with the *mFA-mRuby3* construct. Four hours after transfection we transferred the cells to DMEM base medium or calcification medium for 3 days to mimic kidney calcification. Figure 7C shows mFA-mRuby3-transfected HEK293 cells cultured in DMEM. Like the CHO cells (Figure 1C), mFA-mRuby3-expressing cells were bright red. Figure 7D shows mFA-mRuby3-transfected HEK293 cells cultured in calcification medium. Comparing the area of fluorescence in Figures 7C and D, we determined two striking features separating non-calcifying cells (Figure 7C) from calcifying cells (Figure 7D). First, the total area of fluorescence picked by a threshold increased from 2.5% to 20.3%, suggesting that more cells were stained positive than had expressed mFA-mRuby3. Next, the analysis of size distributions of fluorescent areas showed an increase in the average fluorescent region size from 78 µm^2^ to 142 µm^2^ in non-calcified and calcified cells, respectively. This increase was due to multiple coalescent calcified lesions on the cell monolayer (Figure 7D), similar to the post-stained lesions of non-transfected calcifying cells shown in Figure 7B. A typical image analysis of Figures 7C, D depicted in Figure S4 indicated that calcifying conditions increased the number of fluorescent areas representing single cells or coalesced lesions per field of view from 151 to 377. The cells covered a total area of 2.8 and 12.7% of the view field, respectively, suggesting that at least 2.5-fold more cells were calcified than produced mFA-mRuby3. Concomitantly, calcification conditions increased the average size of the fluorescent areas from 78 to 143 µm^2^ with many areas measuring 500-2,700 µm^2^ suggesting that calcifying HEK293 cells had coalesced into large, calcified lesions. These results indicate that endogenous expression of fluorescent fetuin-A fusion proteins in calcifying cells allowed live, non-toxic detection of calcification in real-time with high specificity, sensitivity, and spatial resolution.

## Discussion

Ectopic calcification poses a significant clinical challenge that currently lacks effective diagnostic and treatment options 31. Previously we demonstrated the use of fluorescence labeled fetuin-A, a major circulating inhibitor of calcification, for the detection of microcalcifications in mouse tissues 13 and in cell culture 32. Here, we further enhanced fluorescent fetuin-A staining with genetically labeled derivates. We expressed in CHO cells intrinsically fluorescent proteins for live detection of microcalcifications in cell cultures. To this end, murine fetuin-A was coupled by a flexible (GGSG)_5_ linker to either a green or red fluorescent protein variant, mEmerald or mRuby3, respectively. The rationale behind this concept was that both GFP and RFP are naturally occurring proteins with low toxicity 33 that could enable live imaging of fine calcifications targeted by fetuin-A. The absence of cellular toxicity of the expressed fusion proteins was validated in mesenchymal stromal cells that revealed no differences in cell viability in cells treated or not with chemically labeled bovine fetuin-A (bFA-AF488), mFA-mEmerald, mFA-mRuby3 (Figure S1). During extended live imaging mFA-mRuby3 detected microcalcifications at 0.23 µM concentrations (Figure 6). At these low concentrations, fetuin-A-based fluorescent probes did not inhibit calcium phosphate precipitation (Figure 2) and therefore did not prevent cell mineralization or calcification even though fetuin-A is a potent inhibitor of mineralization at the physiological 10 µM concentrations. Together with the absence of cellular toxicity (Figure S1), this justified the addition of fluorescent fetuin-A to culture media for extended live imaging of actively mineralizing or calcifying cell cultures.

Recombinant proteins overexpressed in CHO cells, which are optimized for high-yield protein production, partially lack post-translational modifications glycosylation and phosphorylation compared to the natural state. Phosphorylation is, however, critical for the function of many extracellular proteins including those constituting bone and tooth extracellular matrix 34. Therefore, we sought to optimize fetuin-A mineral binding by mimicking the fully phosphorylated state. To this end, we replaced putative Ser and Thr phosphorylation sites with negatively charged glutamic acid residues. Our recent structural work suggested that Ser/Thr phosphorylation in the CTR domain of fetuin-A may enhance interaction with the calcium-rich phases of calcium phosphates. Thus, we chose phosphomimetic amino acid substitutions of serine or threonine residues at positions 135, 138, 305, 309, 312 and 314 with glutamic acid, mimicking the phosphorylated state of fetuin-A. pm-fetuin-A indeed proved more potent in preventing the precipitation of calcium phosphate from supersaturated solutions. Phosphomimetic amino acid exchange restored precipitation inhibition in fetuin-A that had lost mineral binding, most likely because the positively charged HIS-tag located at the C-terminus in the absence of pm-residues unfavorably interacted with the overall negatively charged mineral binding site. Likewise, mutating the putative Ser/Thr phosphorylation sites to glutamic acid in pm-mFA-mEmerald rescued the precipitation inhibition. Phosphomimetic substitutions also increased the inhibitory potential of pm-mFA-mRuby3, even if the IC_50_ shift was small, because the unmodified protein inhibited well initially. Collectively, these data show that phosphomimetic fetuin-A variants have improved calcium phosphate precipitation inhibition, most likely because they better bind and stabilize nascent calcium phosphate mineral. This renders recombinant phosphomimetic fetuin-A variants ideal probes to detect nascent forms of calcium phosphate mineral, which should be abundant in the early stages of calcification. This is important because traditional stains von Kossa or Alizarin Red 35 cannot be used for live staining and, in addition, have variable staining patterns on mineralized tissues. The commercially available fluorescent mineral stain calcein can also be used for live staining but requires large and mature crystalline mineral for binding, precluding its use for nascent calcifications. The same applies to a popular bisphosphonate-based fluorescent probe Osteosense® 36.

The detection capability of the fluorescent fetuin-A variants was tested in cell cultures representing physiological bone mineralization and pathological vascular calcification. Mineralizing osteoblasts generated from mesenchymal stromal cells served as a model for bone mineralization 37. Mineralization was achieved by employing established osteogenic medium containing dexamethasone, ascorbic acid and beta-glycerophosphate as an organic phosphate source, but without elevated calcium and phosphate 38, 39. The cells mineralized their extracellular matrix resulting in homogenous sand grain-like mineralization that demarcated the cell outline. Calcification of VSMC served as a model of pathological vascular calcification, a hallmark of chronic kidney disease, which is associated with elevated extracellular phosphate. We triggered iVSMC calcification by increasing calcium and phosphate in the culture medium at concentrations insufficient for spontaneous mineral precipitation in the absence of cells. Although the mineralized MSC-derived osteoblasts and calcified iVSMC look similar when stained with Alizarin Red S, the fluorescent fetuin-A probes clearly differentiated two distinctive calcification patterns. Unlike the cell delineating mineralization observed in MSC, calcification of iVSMC was strongest at sites away from nucleated cells (Figure 4), suggesting staining of predominantly cell remnants of perished cells and thus pathological dystrophic calcification. Our data support the notion that elevated calcium and phosphate are toxic in VSMC 40, 41. The finding that VSMC first perish and then calcify also supports the observation that severe vascular calcification in CKD patients is associated with loss of VSMC 42. Although we observed a strong correlation between the staining patterns of fluorescent fetuin-A and calcein in severely calcified iVSMC, this was not the case for MSC cultures, where calcein failed to stain the finely grained extracellular matrix mineralization.

The unique ability of a fetuin-A molecule to preferentially bind to the nascent mineral was used to specifically stain small, calcified lesions in kidney tissue from calcification-prone mice. Both chemically labeled fluorescent fetuin-A and genetically labeled fluorescent fetuin-A readily detected microcalcifications and demarcated large, calcified tissue predominantly at their periphery giving the appearance of “empty” lesions. Again, this suggested that fetuin preferentially binds nascent calcium phosphate mineral comprising small lesions and the surface of large lesions. Staining of identical sections with Alizarin Red S and calcein left most of the smaller calcified lesions unstained, supporting the specificity and sensitivity of fetuin-A for small nascent calcifications. Table S1 compares established calcification staining with fetuin-A-based staining. Application of a fetuin-A fluorescent probe for the first time also revealed areas of calcifications in the luminal spaces of the proximal convoluted tubules consistent with the histopathological profile of intratubular nephrocalcinosis 43, 44. This finding is in line with the present understanding of calcium reabsorption in nephrons 45. The sensitivity of Alizarin Red S staining was insufficient to identify the described features. This finding justifies the use of fluorescent fetuin-A as a diagnostic probe in kidney disease, as the organ is known to undergo minimal histological changes unresolvable using conventional microscopy 46. Given that the proximal convoluted tubules are functionally active segments of the nephron responsible for the reabsorption of the major part of the glomerular ultrafiltrate, intraluminal tubular calcification may strongly affect kidney function. Indeed, there is a solid body of evidence showing that nephrocalcinosis may accelerate the progression of CKD 43. Thus, application of fetuin-A-based fluorescent probes can become a quick and cost-effective method for the detection of early-stage kidney calcifications.

To compare exogenous with endogenous fluorescent fetuin-A staining, we performed extended live imaging of HEK293 kidney epithelial cells which expressed mFA-mRuby3. Fluorescence imaging revealed the mFA-mRuby3 expressing cells in non-calcifying conditions and in addition, a much larger number of mFA-mRuby3 stained cells in calcifying conditions. Although the intracellular signal of mFA-mRuby3-expressing cells was stronger than that derived from calcified zones, both were readily distinguishable due to the typical spherical morphology of the transfected cells. The amount of the fluorescent protein contained in medium 3 days post-transfection was sufficient to also stain calcified matter in neighboring untransfected cells and cell aggregates. These findings validate endogenous mFA-mRuby3 expression as a useful cell-based calcification reporter system that may be further developed into stably expressing cells to be tested alone or in combination with other cell cultures.

We are aware that our research has several limitations. Major modifications of protein structure, as well as fusion protein production, often lead to a loss of function. One of the two chimeric proteins with seemingly similar structures used in this study had impaired activity that was rectified by phosphomimetic substitution of several serine/threonine phosphorylation sites. Recent advances in the prediction of protein structure and folding may partially address this problem in the future 21. Nevertheless, fluorescent fusion proteins require further optimization before being used as diagnostic probes *in vivo*. Despite high sensitivity and specificity toward microcalcifications, the fetuin-A-based fluorescent probes are not perfectly suited for *in vivo* imaging as they emit light of the visible spectrum, which is readily absorbed by hemoglobin and other endogenous fluorescence quenching molecules 47. We suggest improving probes by attaching fluorescent proteins absorbing and emitting in a near-infrared (~750-900 nm) optical window where light penetrates deeper into tissues and scatters less 48. Moreover, the C-terminal polyhistidine tag used for protein purification may also be used for radiolabeling of the fluorescent probes for combined fluorescent and nuclear imaging 49 as well as coupling biologically small molecules to develop fetuin-A-based probes into targeted theranostics of calcification.

## Conclusion

Ectopic microcalcifications represent a major clinical concern as they trigger inflammation and a vicious cycle of tissue degeneration and loss of function. Due to their small size, they are hard to detect using conventional imaging techniques. Here, we describe novel fetuin-A-based fluorescent probes for imaging microcalcifications with high sensitivity and specificity. We show that chimeric proteins consisting of a fetuin-A mineral binding moiety and a fluorescent protein moiety surpass existing methods for detecting small and nascent calcified lesions. Good cytocompatibility and safety profile of fluorescent fetuin-A proteins allow extended live imaging of calcifying cells and the development of cell-based calcification reporter platforms. Compatibility of the produced fluorescent probes with advanced fluorescent imaging modalities including TPE microscopy renders fetuin-A-based fluorescent proteins promising probes for the non-invasive detection of calcifications *in vivo*.

## Supplementary Material

Supplementary figures and table.

## Abbreviations

Ahsg: α2-Heremans-Schmid glycoprotein
bFA: bovine fetuin-A
CHO: Chinese hamster ovary
CKD: chronic kidney disease
CPP: calciprotein particle
CTR: C-terminal region
CY1: cystatin-like domain 1
DMEM: Dulbecco's Modified Eagle's Medium
FBS: fetal bovine serum
HEK293: human embryonic kidney 293
IMAC: immobilized metal affinity chromatography
iVSMC: immortalized vascular smooth muscle cells
LANUV: Landesamt für Natur-, Umwelt- und Verbraucherschutz
mFA: murine fetuin-A
MSC: mesenchymal stromal cells
PBS: phosphate-buffered saline
PCC: Pearson correlation coefficient
TPE: two-photon excitation
VSMC: vascular smooth muscle cells
