# Supplementary material for: Application of the mineral-binding protein fetuin-A for the detection of calcified lesions
Source: Theranostics. 2023 Jan 1;13(2):659–72. doi: 10.7150/thno.78773 (PMC9830449; doi:10.7150/thno.78773)
Supplement: Supplementary file 1 — Supplementary figures and table. [file thnov13p0659s1.pdf]

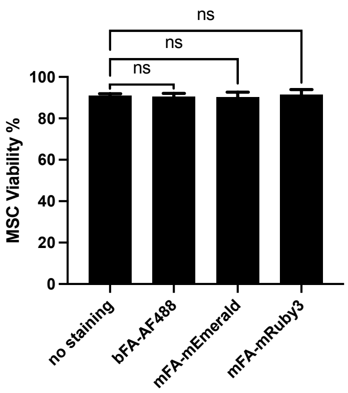

Figure S1. Fluorescent Fetuin-A proteins are non-toxic in mesenchymal stromal cells (MSC). Viability was tested by Trypan Blue dye exclusion 72 h after a 2 h incubation with the indicated fluorescent fetuin-A derivatives. Cells were treated in triplicated wells of a 24-well plate. A minimum of 400 cells was counted in each well. Values represent mean ± SE, n=3.

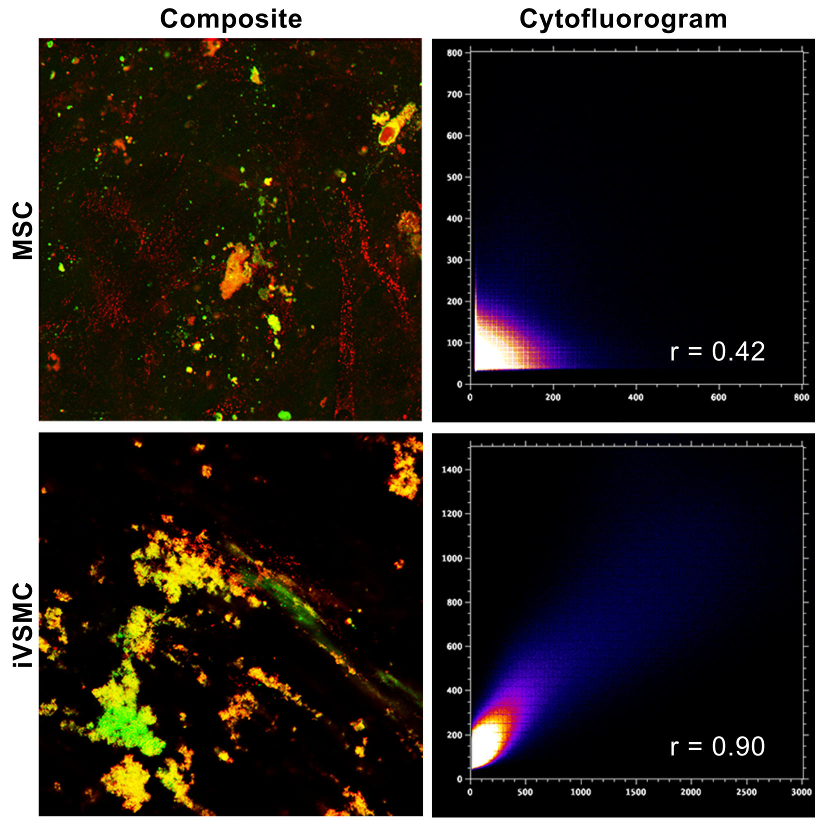

Figure S2. Composite images and co-localization analysis of mineral deposits in mFA-mRuby3- (left panel, red) and calcein-stained cells (left panel, green). Right panel: corresponding cytofluorograms. The intensity of pixel fluorescence increase is shown using a heatmap. r-value: the Pearson correlation coefficient.

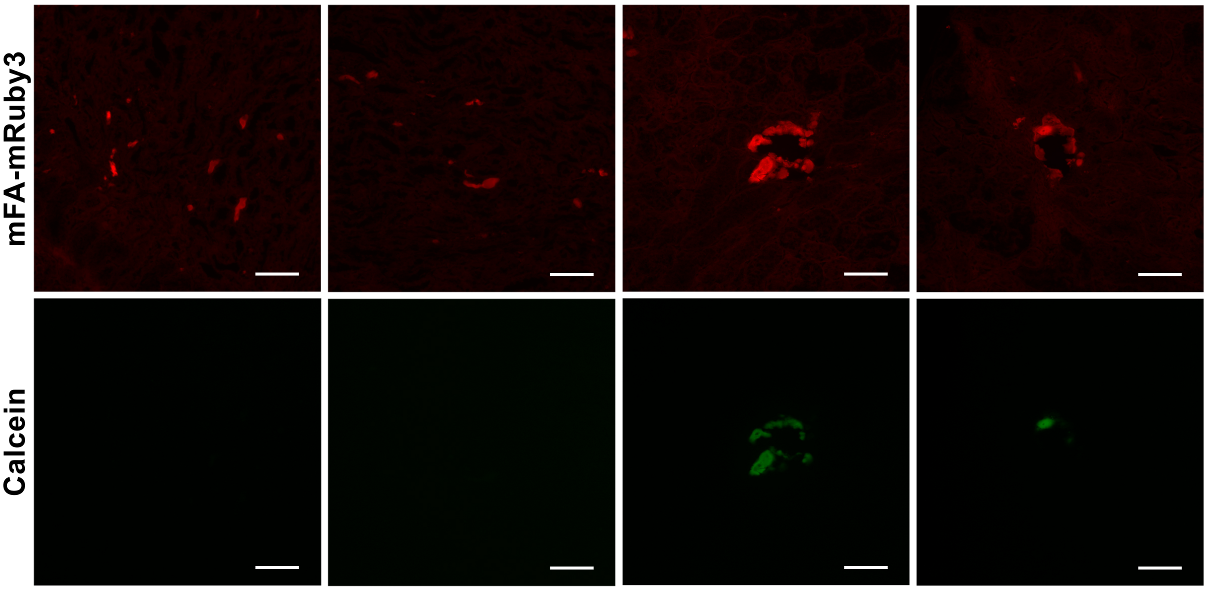

Figure S3. Simultaneous staining of kidney calcifications with mFA-mRuby3 and calcein. Upper panel: mFA-mRuby3-derived signal (red). Lower panel: calcein-derived fluorescence (green). Scale bars 100 µm.

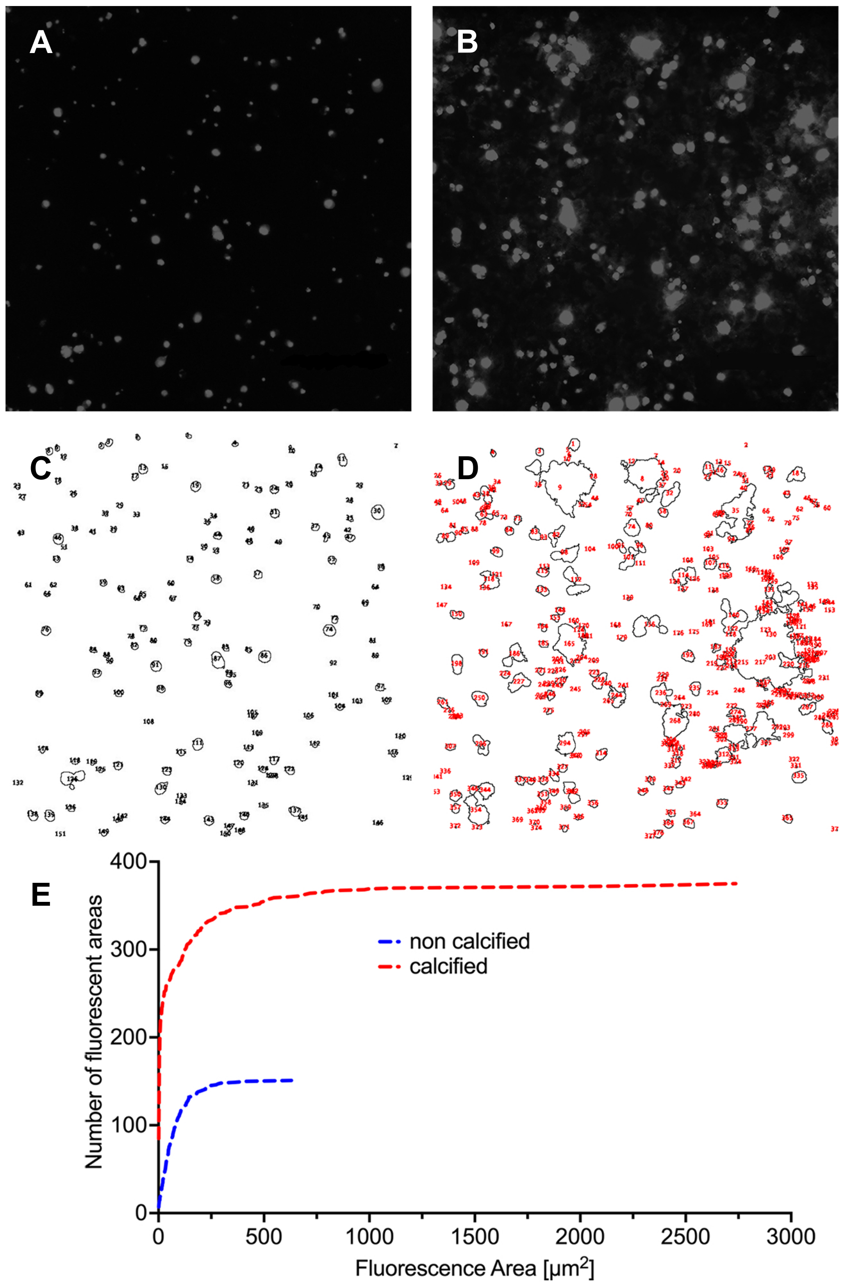

Figure S4. Analysis of calcified areas depicted in Figures 7C, D. **(A, B)** Typical micrographs were converted to 8-bit grayscale images. **(C, D)** Particle analysis was performed with a threshold set manually to match the contours of fluorescent areas. **(E)** Accumulate frequency distribution of areas identified in C and D showing that both the number and the size of areas increased in calcified HEK293 cells expressing mFA-mRuby3.

Table S1. Comparison of stains used for histopathological assessment of calcification.

**Stain**	**Chemical composition**	**Sensitivity**	**Specificity**	**Suitability for histological staining**	**Suitability for live imaging**	**Cell reporter system**
Alizarin Red S	Alizarin sulfonic acid sodium salt	+	+ (binds carbonates)	yes	no (requires fixation)	no
Calcein	Fluorescein complex	++	+ (binds Cu^{++}, Zn^{++}, Al^{++})	yes	yes	no
bFA-AF488	bovine fetuin-A labeled with Alexa Fluor™ 488 NHS ester	+++	binds nascent apatite	yes	yes	no
pm-mFA-mEmerald	murine fetuin-A coupled to a GFP derivative	+++	binds nascent apatite	yes	yes	yes
pm-mFA-mRuby3	murine fetuin-A coupled to an GFP derivative	+++	binds nascent apatite	yes	yes	yes
